# Highlight report: The pseudolobule in liver fibrosis

**DOI:** 10.17179/excli2017-1038

**Published:** 2017-12-20

**Authors:** H.M. Bolt

**Affiliations:** 1 IfADo, Leibniz Research Centre for Working Environment and Human Factors, Dortmund

## ⁯⁯

Recently, Seddik Hammad from Heidelberg University published an interesting report about a frequent misinterpretation in research on liver fibrosis (Hammad et al., 2017[9]): in mice repeated doses of carbontetrachloride (CCl_4_) cause a pattern of fibrosis, in which pseudolobules occur that are lined by fibrotic streets, which can be visualized by Sirius red staining. 

In the center of these pseudolobules vessels can be seen that have been interpreted as central veins. Although the perception that the vessel in the center of the pseudolobule is a central vein may seem intuitively understandable, this clearly represents a misinterpretation. In reality, the vessel in the center of the pseudolobule is a portal vein. In contrast, the central veins are found within the fibrotic streets. This clarification could be achieved by the use of previously established markers that exclusively stain the hepatocytes around the central vein and by specific periportal markers (Hammad et al., 2014[10]). Hammad and colleagues explain the mechanism responsible for this pattern by CCl_4_ mediated pericentral killing of CYP2E1 positive hepatocytes, which after repeated CCl_4_ administration leads to fibrotic bridging of pericentral areas (Hammad et al., 2017[9]).

Studies of hepatotoxicity often rely on the correct interpretation of histology (Schenk et al., 2017[18]; Reif et al., 2017[17]; Ghallab et al., 2016[8]; Vartak et al., 2016[20]; Nussler et al., 2014[16]; Drasdo et al., 2014[6]; Campos et al., 2014[3]; Braeuning and Schwarz, 2016[2]; Chen et al., 2015[4]; Crespo Yanguas et al., 2016[5]). Also liver physiology and regeneration depend on optimal zonation (Jansen et al., 2017[12]; Hoehme et al., 2010[11]; Bartl et al., 2015[1]; Yanguas et al., 2016[21]; Stöber, 2016[19]; Moghbel et al., 2016[15]): moreover 3D *in vitro* systems in toxicology aim for mimicking some of the zonated features of the liver lobule (Frey et al., 2014[7]; Kim et al., 2015[13]; Leist et al., 2017[14]). Therefore, the careful analysis of Hammad and colleagues may help to avoid some misunderstanding in future.
